# The Sultan’s Food

**Published:** 1893-01

**Authors:** 


### THE SULTAN'S FOOD.

The Sultan of Turkey leads a very simple life. He came to the throne in 1876, without any agency cf his own, and almost against his own will, after living for many years in retirement, and no doubt finds the trappings of royalty something of a burden.

When it is said that he lives simply, however, the word must be understood as applying to his personal habits rather than to his official surroundings and expenditures. Thus it is estimated that more than six thousand persons are fed every day at his Dolma Bagtche palace when he is there. The treasurer of the household has a pretty heavy burden upon his shoulders.

There is a regularly organized force of buyers, each charged with the purchase of certain supplies for the palace. One man's duty is to buy fish, and to do this for six thousand persons is no light undertaking in a city which has no great markets. About ten tons a week are required, and to secure this some twenty men are kept busy.

Nearly eighteen thousand pounds of bread are eaten daily, and all this is baked in enormous ovens at some distance from the palace. Of course a large force of bakers is required, as well as another large force of buyers and carriers of flour and fuel.

The Sultan's own food is prepared by one man and his assistants, and no others touch it. It is cooked in silver vessels, and when done, each kettle is sealed by a slip of paper and a stamp. This stamp is broken in the presence of the Sultan by the High Chamberlain, who takes one spoonful of each kettle before the Sultan tastes it—as a safeguard against poison.

Nearly a ton of rice a day is required for the inevitable *pilaff*, together with six hundred pounds of sugar, and an equal amount of coffee, to say nothing of the other groceries, fruit, vegetables and meat.

That there is enormous waste and extravagance in the kitchens is almost a matter of course ; it is said that enough is thrown away daily to feed a hundred families. But such waste is not confined to a Turkish royal household, and might be found in kitchens nearer home. The surplus is gathered up by the beggars, with whom Constantinople abounds, and what still remains is eaten by the scavenger dogs.

